# Scale-up bioprocess development for production of the antibiotic valinomycin in *Escherichia coli* based on consistent fed-batch cultivations

**DOI:** 10.1186/s12934-015-0272-y

**Published:** 2015-06-12

**Authors:** Jian Li, Jennifer Jaitzig, Ping Lu, Roderich D Süssmuth, Peter Neubauer

**Affiliations:** Chair of Bioprocess Engineering, Department of Biotechnology, Technische Universität Berlin, Ackerstraße 76, ACK24, 13355 Berlin, Germany; Department of Chemistry, Technische Universität Berlin, Straße des 17. Juni 124, 10623 Berlin, Germany; Department of Chemical and Biological Engineering, Northwestern University, 2145 Sheridan Road, Evanston, IL 60208 USA

**Keywords:** Valinomycin, Synthetic biology, Bioprocess development, Scale-up, Fed-batch, *Escherichia coli*

## Abstract

**Background:**

Heterologous production of natural products in *Escherichia coli* has emerged as an attractive strategy to obtain molecules of interest. Although technically feasible most of them are still constrained to laboratory scale production. Therefore, it is necessary to develop reasonable scale-up strategies for bioprocesses aiming at the overproduction of targeted natural products under industrial scale conditions. To this end, we used the production of the antibiotic valinomycin in *E. coli* as a model system for scalable bioprocess development based on consistent fed-batch cultivations.

**Results:**

In this work, the glucose limited fed-batch strategy based on pure mineral salt medium was used throughout all scales for valinomycin production. The optimal glucose feed rate was initially detected by the use of a biocatalytically controlled glucose release (EnBase® technology) in parallel cultivations in 24-well plates with continuous monitoring of pH and dissolved oxygen. These results were confirmed in shake flasks, where the accumulation of valinomycin was highest when the specific growth rate decreased below 0.1 h^−1^. This correlation was also observed for high cell density fed-batch cultivations in a lab-scale bioreactor. The bioreactor fermentation produced valinomycin with titers of more than 2 mg L^−1^ based on the feeding of a concentrated glucose solution. Valinomycin production was not affected by oscillating conditions (i.e. glucose and oxygen) in a scale-down two-compartment reactor, which could mimic similar situations in industrial bioreactors, suggesting that the process is very robust and a scaling of the process to a larger industrial scale appears a realistic scenario.

**Conclusions:**

Valinomycin production was scaled up from mL volumes to 10 L with consistent use of the fed-batch technology. This work presents a robust and reliable approach for scalable bioprocess development and represents an example for the consistent development of a process for a heterologously expressed natural product towards the industrial scale.

## Background

Natural products are of great significance because they are important sources for pharmaceuticals to treat human and animal diseases [1]. These natural compounds include nonribosomal peptides (NRPs, e.g. vancomycin), polyketides (PKs, e.g. erythromycin) and their hybrids (NRP/PKs, e.g. epothilone). In recent years, a large number of NRPs and PKs have been discovered by genome mining [2], however, their potential further development and clinical application are often hampered due to the fact that their native producers are not cultivable. Alternatively, with the development of synthetic biology, some NRPs and PKs have been synthesized by heterologous expression of their biosynthetic gene clusters in several well-characterized and genetically tractable host microorganisms [3–12]. While multiple options for a surrogate host exist, *Escherichia coli* has been chosen as a highly promising and robust cell factory for heterologous production of NRPs and PKs, given its versatile advantages including easy cultivation, genetic manipulation and scalable fermentation [13].

**Figure 1:**
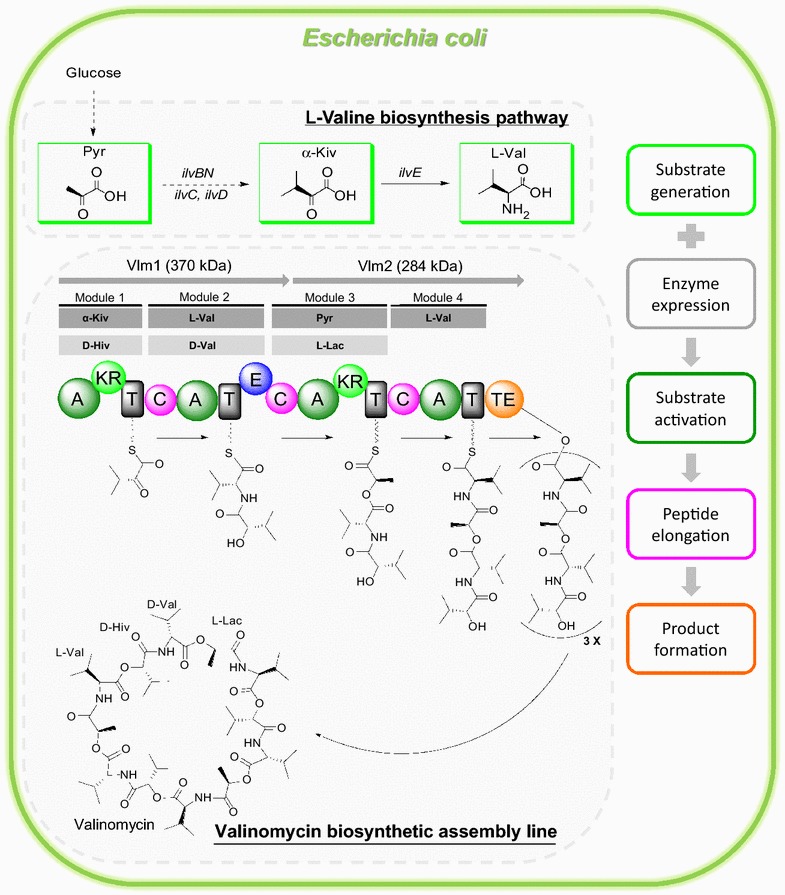
Proposed valinomycin biosynthetic pathway in the host *E. coli*. *Pyr* pyruvate, l *-Lac* lactate, *α-Kiv* α-ketoisovalerate, d *-Hiv* d-hydroxyisovalerate, l *-Val* l-valine, d *-Val* d-valine, *ilvBN* acetohydroxy acid synthase I, *ilvC* acetohydroxy acid isomeroreductase, *ilvD* dihydroxy acid dehydratase, *ilvE* branched chain amino acid aminotransferase, *A* adenylation domain, *T* thiolation domain, *C* condensation domain, *KR* ketoreductase domain, *E* epimerase domain, *TE* thioesterase domain. A domains select and activate the substrates. T domains are responsible for the translocation of the bound aminoacyl or peptidyl intermediate between adjacent catalytic positions. C domains catalyze the formation of peptide bond and elongate the peptide chain. The KR domain in module 1 reduces α-Kiv to d-Hiv. The E domain in module 2 transfers l-Val to d-Val. The KR domain in module 3 reduces Pyr to l-Lac. The four modules of valinomycin synthetase are iteratively reused to assemble three tetradepsipeptide monomers, which are eventually oligomerized and macrolactonized to form the cyclododecadepsipeptide valinomycin.

Valinomycin is an NRP cyclodepsipeptide antibiotic that is naturally biosynthesized by several *Streptomyces* strains via the enzyme valinomycin synthetase, which belongs to the mRNA-independent assembly lines termed nonribosomal peptide synthetase (NRPS) [14–16]. Valinomycin synthetase contains two distinct large NRPSs, Vlm1 (370 kDa) and Vlm2 (284 kDa), and each of them has two modules to assemble their dedicated substrates α-ketoisovaleric acid (module 1), l-valine (modules 2 and 4) and pyruvate (module 3) (for a biosynthetic model see Figure 1). To realize heterologous production of valinomycin, we have successfully reconstituted the NRPS genes (*vlm1* and *vlm2*) from *Streptomyces tsusimaensis* in an engineered *E. coli* host [17]. Autonomous formation of valinomycin could be achieved without substrate feeding, because the three substrates are available from the glycolysis as well as from the branched chain amino acid l-valine biosynthesis pathway in *E. coli* (Figure 1). The initial valinomycin titer was modest (0.3 mg L^−1^) in batch cultivations, while the titer was significantly enhanced up to 10 mg L^−1^ through flask scale high cell density fed-batch cultivations [18]. In addition, coexpression of the cognate type II thioesterase (TEII), which is encoded in the valinomycin gene cluster and responsible for regeneration of the NRPS activity, with Vlm1 and Vlm2 further improved valinomycin production to a final titer of 13 mg L^−1^ [19].

Although heterologous production of some NRPs and PKs in *E. coli* has been achieved over the last decade, only a few of them have been produced in large-scale bioreactors, yet the yields were mostly not satisfactory [13]. The polyketide 6-deoxyerythronolide B (6-dEB, precursor of the antibiotic erythromycin) was intensively investigated as a model system. Up to now, the reported highest titer of 6-dEB was 1.1 g L^−1^, achieving through a 12-day high cell density fed-batch bioreactor fermentation [20]. However, the final compound erythromycin A just reached a titer of 4 mg L^−1^ in the bioreactor with a batch mode cultivation for 6 days [21]. The antitumor compound echinomycin was the first NRP that was heterologously produced in *E. coli* with a titer of 0.3 mg L^−1^ after 8 days of fed-batch fermentation [22]. All studies show that, for increased yields of the target NRPs or PKs, it is challenging not only to recombinantly express the large biosynthetic enzymes, but also to maintain the fitness of the cells over comparably long times of cultivation to guarantee the continuous production of the substrate metabolites. Therefore, more efforts, especially in the context of fed-batch and scale-up bioprocess development, need to be done.

Generally, prior to scale up a bioprocess, the parameters are initially optimized through small scale batch cultivations. However, the optimized parameters might not be transferable directly from small batch to large fed-batch fermentations because of the different cultivation modes. Recently, the enzyme-controlled glucose delivery EnBase® strategy has been developed to cultivate microbial cells under a fed-batch type of cultivation in small-scale vessels such as shake flasks [23]. The EnBase technology enables scalability from microwell plates to larger bioreactors with consistent conditions for the production of different recombinant proteins [24, 25]. While consistent cultivations can be solved by the EnBase system, inhomogeneity is another main concern during the industrial scale fermentations due to the limited mass transfer. An important feature of different product yields in laboratory and industrial scale is especially the existence of a feeding zone which is characterized by a high glucose concentration [26]. As a high glucose concentration is connected to a high metabolic activity, also oxygen limitation appears in such a zone at the typical high cell concentrations [27]. In the case of production of small molecules such a feeding zone directly affects the flux through the central metabolic pathways [28] and thus may influence the yield of the product. To address this problem, a laboratory scale-down two-compartment reactor (TCR) was applied to simulate the effect of the feed zone and thus to study the robustness of the process [29]. The metabolic and physiological responses of microbial cells in the TCR cultivation environment could thus reflect the situations under a very similar industrial production process, generating useful and valuable information for bioprocess development.

**Figure 2:**
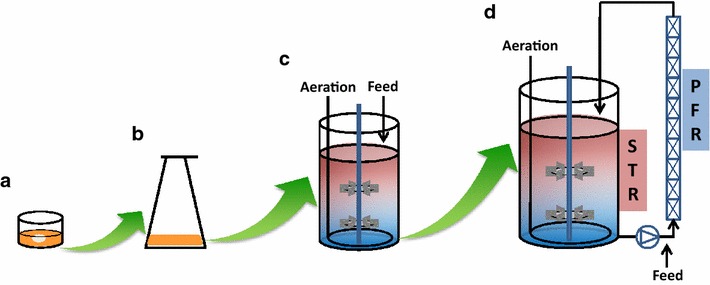
Scale-up bioprocess development for valinomycin production in *E. coli* from 1 mL to 10 L. **a** A representative well from the 24-well plates, which are incorporated with sensors allowing online measurement of oxygen and pH. Cultivations were performed in 24-well plates with 1 mL working volume per well (total volume 3.3 mL); **b** cultivations were performed in 500 mL shake flasks with 100 mL working volume; **c** cultivation was performed in a 3.7 L bioreactor with 2 L working volume; **d** cultivations were performed in a 15 L scale-down TCR with 10 L working volume. *STR* stirred tank reactor, *PFR* plug flow reactor.

In this work, we aim to develop a rational scale-up bioprocess route at the example of valinomycin production (see Figure 2 for the scale-up scheme from 1 mL to 10 L), which we believe provides a reasonable bioprocess development approach for realizing natural product production to meet the demand.

## Methods

### Bacterial strains and culture medium

The host *E. coli* strain BJJ01 [F^−^*ompT hsdS(r*_*B*_^−^*m*_*B*_^−^*) dcm*^+^ Tet^R^*gal endA* Hte *ΔxylA::sfp*_*wt*_] is a derivative of *E. coli* BL21 Gold (Agilent Technologies, Waldbronn, Germany) [17]. It contains *sfp* gene from *Bacillus subtilis* for posttranslational phosphopantetheinylation of NRPSs chromosomally integrated into the *xylA* locus. Two valinomycin producing strains, *E. coli* BJJ01 pCTUT7-Vlm1 pKS01-Vlm2 that expresses valinomycin synthetase Vlm1 and Vlm2 [17] and *E. coli* BJJ01 pJL03-Vlm1 pJL07-Vlm2 pJL10-TEII that expresses Vlm1, Vlm2 and a third repairing enzyme type II thioesterase [19], were used in this study. EnPresso B defined medium without any complex nutrients (BioSilta, Cambridge, UK) was used for valinomycin production in 24-well plates and shake flasks, and as a starter medium in the large bioreactors. The monomer glucose is gradually released from the glucose polymer by a biocatalyst (Reagent A, BioSilta) to feed cells during the cultivation.

### Cultivation in 24-well plates

To prepare a preculture, *E. coli* cells were inoculated into 100 mL EnPresso B defined medium in 500 mL Ultra Yield Flasks™ (Thomson Instrument Company, CA, USA), which was supplemented with 0.3 U L^−1^ glucose-releasing biocatalyst, 10 μL antifoam 204 (Sigma) and appropriate antibiotics (17 mg L^−1^ chloramphenicol and 50 mg L^−1^ ampicillin). The flask was incubated overnight on an orbital shaker (amplitude: 25 mm, Infors HT, Switzerland) at 200 rpm and 30°C. Afterwards, the overnight preculture was distributed without diluting it into the 24-well plates (PreSens Precision Sensing GmbH, Regensburg, Germany) with 1 mL per well. To screen the optimal biocatalyst concentration, additionally to the initial 0.3 U L^−1^ further amounts of biocatalyst were added to each well (1.5, 3, 9 or 15 U L^−1^). The 24-well plates were covered with the Duetz System sandwich cover and clamp system (Enzyscreen, Er Haarlem, The Netherlands) for efficient oxygen transfer and low evaporation (appr. 0.1 mL per well over the cultivation time). The subsequent cultivation was continued for 48 h at 30°C and 250 rpm in a shaker (amplitude: 50 mm, Kühner, Birsfelden, Switzerland) with online measurement of DO and pH (SensorDish Reader, PreSens).

### Shake flask cultivation

Shake flask cultivations were performed in 500 mL Ultra Yield Flasks with membrane seals (both from Thomson Instrument Company, CA, USA) containing 100 mL EnPresso B defined medium with inoculation of OD_600_ at 0.1. A biocatalyst concentration of 3 U L^−1^ to release glucose was added at the start of the experiments. The cultivations were carried out at 200 rpm and 30°C for 12 h in a shaker (amplitude: 25 mm, Infors HT, Switzerland).

### Bioreactor fed-batch fermentation

The high cell density fed-batch fermentation for valinomycin production was performed in a 3.7 L bench-top bioreactor (KLF2000, Bioengineering, Switzerland). The cultivation comprised two phases: (1) initial cultivation in EnPresso B defined medium and (2) a fed-batch cultivation with external glucose feeding (a concentrated feed solution contained 400 g L^−1^ glucose). 2 L EnPresso B as a starter medium were prepared without complex nutrient additives and autoclaved in the bioreactor at 121°C for 20 min. 3 U L^−1^ biocatalyst (Reagent A, BioSilta) were added to the medium directly before inoculation with an initial OD_600_ of 0.1. Cells were grown at 30°C and the pH was maintained at 7.0 with 25% NH_4_OH and 10% H_3_PO_4_. The level of DO was maintained above 20% (air saturation) through adjustment of the stirrer rate and air flow rate during the fermentation. Foaming was controlled by adding 2 mL antifoam (PPG2000) per time manually when foam appeared.

When the glucose releasing polymer in the EnPresso B medium was depleted 16–18 h after inoculation (as indicated by zero residual glucose), external glucose feeding was initiated at a rate of 0.07 mL min^−1^, which is calculated according to the following equation:

$$F_{0} { = }\frac{{\mu X_{0} V_{0} }}{{S_{f} Y_{X/S} }}$$

where *X*_0_ and *V*_0_ are the cell dry weight (g L^−1^) and culture volume (L) at the time of feeding start, *μ* is the specific growth rate (0.2 h^−1^), *S*_*f*_ is the glucose concentration in the feeding solution (400 g L^−1^), and *Y*_*X/S*_ is the yield coefficient (0.4 g g^−1^, biomass produced per glucose).

The subsequent exponential feeding process was continued for 12 h with a specific growth rate (*μ*) of 0.2 h^−1^ and the feeding profile was calculated according to the following equation:

$${\text{F}}\left( t \right) = F_{0} \cdot e^{ \mu \cdot t}$$

where *F*_0_ is the initial feeding rate (L h^−1^), *µ* is the specific growth rate (h^−1^) to be maintained, and *t* is the time (h) after feeding start.

Afterwards, the glucose feeding rate was gradually decreased from 0.85 to 0.45 mL min^−1^ in 6 h to prevent the DO to drop under 20% in the bioreactor, followed by constant feeding at 0.45 mL min^−1^ until the end of the fermentation. During the whole fed-batch phase, when OD_600_ increased by ~20, a mixture of 2 mL MgSO_4_ (1.5 M), 2 mL trace element solution [per L: 0.5 g CaCl_2_∙2H_2_O, 0.18 g ZnSO_4_∙7H_2_O, 0.10 g MnSO_4_∙H_2_O, 20.1 g Na_2_-EDTA, 16.70 g FeCl_3_∙6H_2_O, 0.16 g CuSO_4_∙5H_2_O, 0.18 g CoCl_2_∙6H_2_O, 0.132 g Na_2_SeO_3_∙5H_2_O, 0.12 g Na_2_MoO_4_∙2H_2_O, and 0.725 g Ni(NO_3_)_2_∙6H_2_O], and 2 mL thiamine (50 g L^−1^) was added aseptically to the bioreactor containing 2 L culture. Samples were drawn from the bioreactor for OD_600_ measurement, residual glucose determination and valinomycin quantification.

### Two-compartment reactor fermentation

To investigate valinomycin production under oscillating conditions, a scale-down TCR system, which consists of a 15 L standard STR (B. Braun + Diessel Biotech GmbH, Germany) and a 1.2 L PFR equipped with static mixers, was used for the fermentation (see Figure 2d). The strain *E. coli* BJJ01 pJL03-Vlm1 pJL07-Vlm2 pJL10-TEII was used in this experiment. For comparison, one reference fermentation was performed in the STR without PFR. In the reference STR fed-batch fermentation, 10 L EnPresso B defined medium were used for the initial cultivation followed by a glucose fed-batch cultivation. The exponential feeding was continued for 8 h with a specific growth rate (*μ*) of 0.2 h^−1^. In the TCR (STR + PFR) fed-batch fermentation, glucose was fed at the inlet of the PFR with the *μ* of 0.22 h^−1^ for 8 h. The culture was circulated between the STR and the PFR by a pump with a residence time of 1 min in the PFR. The inoculation, cultivation temperature, DO, pH and other salt additives were kept the same as described above in “Bioreactor fed-batch fermentation”. All samples were drawn from the STR for OD_600_ measurement, residual glucose determination and valinomycin quantification.

### Cell growth determination

Cell growth in shake flasks and bioreactors was measured by optical density at 600 nm (OD_600_) with a UV/Vis spectrophotometer (Ultrospec 3300, Amersham Biosciences, Germany). For the 24-well plate samples, cell densities were automatically measured using a robotic platform (Hamilton Robotics, Switzerland) by mixing 5 μL culture with 145 μL of 0.9% NaCl in 96-microwell plates, followed by OD_600_ reading with a Synergy Mx microplate reader (BioTek Instruments, USA). All OD_600_ values were then recalculated for a light path length of 1 cm according to a calibration curve. All measurements were determined in triplicate.

The specific growth rate (*μ*) was calculated for the period between two consecutive OD_600_ measurements and was then fitted with the best spline.

### Glucose determination

Samples were drawn from bioreactor cultivations and centrifuged at 12,000×*g* for 5 min. Residual glucose concentration in the supernatant was determined by the Glucose Hexokinase FS kit (DiaSys Diagnostic Systems GmbH, Holzheim, Germany).

### Valinomycin preparation and analysis

Valinomycin extraction and quantification were carried out as previously described [18]. Briefly, 1 mL of culture was centrifuged at 16,000×*g* for 3 min. The resulting supernatant and cell pellet were extracted each with 2 mL ethyl acetate and 2 mL methanol, respectively. Then the two extracts were combined, vacuum dried, and resuspended in 1 mL of methanol. Valinomycin analysis was performed with an Agilent 6460 Triple Quadrupole LC/MS System using an Eclipse Plus C18 column (RRHD 1.8 μm, 2.1 × 50 mm) by injection of 1 μL sample volume. Separations were achieved at a flow rate of 0.3 mL min^−1^ with elution buffers A (water + 0.1% formic acid) and B (acetonitrile + 0.1% formic acid) through a linear gradient from 5 to 100% B over 2.5 min, an isocratic wash at 100% B for 7.5 min and a linear decrease to 5% B within 2 min. Valinomycin concentrations were calculated according to a calibration curve generated with valinomycin standard (Sigma). All measurements were performed in triplicate.

## Results and discussion

### Cultivations in milliliter scale

Bioprocess development usually starts from small-scale cultivations to evaluate optimal parameters. However, those parameters may not be directly applied to the following large-scale production due to the inconsistent conditions between small and large cultivation modes (e.g. batch vs fed-batch). To circumvent this inconsistency, we used the fed-batch type of cultivation for valinomycin production over the whole process development procedure, which is also necessary to guarantee the long-term production of valinomycin. While in our earlier papers [18, 19], we applied media with complex additives for the production of valinomycin, here we aimed for simple glucose mineral salt based medium, for easier process development. As the building blocks for valinomycin all derive from the central carbon metabolism, a fermentation strategy based on glucose as the sole carbon and energy source should work out for the production of this NRP. An important parameter in the initial screening experiments was the variation of glucose feed rate, which was established by varying the biocatalyst concentrations ranging from 1.5 to 15 U L^−1^.

**Figure 3:**
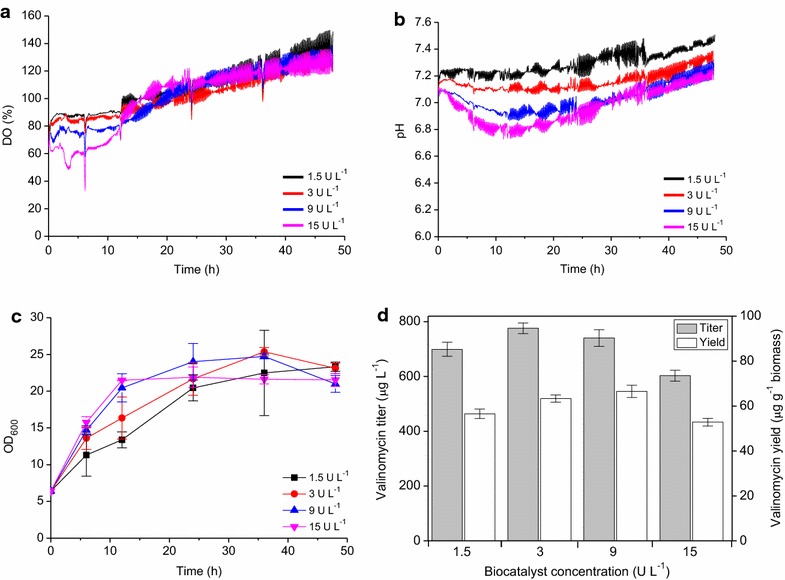
Valinomycin production with different biocatalyst concentrations using EnPresso B defined medium in milliliter-scale cultivations of *E. coli* BJJ01 pCTUT7-Vlm1 pKS01-Vlm2. **a** Dissolved oxygen (DO) and **b** pH were measured online; **c** cell growth curves; **d** valinomycin production. An overnight preculture of *E. coli* BJJ01 pCTUT7-Vlm1 pKS01-Vlm2 (OD_600_ of ~6.0) was distributed without diluting it into the 24-well plates with 1 mL per well (total volume 3.3 mL). Different amounts of biocatalyst (1.5, 3, 9 or 15 U L^−1^) were added to each well followed by 48 h cultivation at 250 rpm and 30°C with online measurement of DO and pH.

Clearly, with more biocatalyst in the culture, a lower dissolved oxygen (DO) level was observed during the first 12 h cultivation (Figure 3a). This is because glucose was released fast with high concentrations of biocatalyst, which makes cells growing rapidly and consuming more oxygen. After 12 h, oxygen contents from the four conditions became similar and gradually increased to the end of the cultivation. The DO increase is likely due to the steadily decreasing amount of the glucose polymer, which also causes the growth rates of the cultures to decline. As shown in Figure 3b, increasing amounts of biocatalyst (9 and 15 U L^−1^) led to a faster decrease of pH, suggesting possible overfeeding and accumulation of typical acidic products of the mixed acid fermentation in the first 12 h of cultivation. By contrast, at the lowest concentration of biocatalyst (1.5 U L^−1^), the pH values were always the highest, indicating that cells suffered a higher degree of starvation from a slower glucose feeding rate. With the biocatalyst concentration of 3 U L^−1^, the pH values were relatively stable at around 7.1 during the entire cultivation, which means that cells could grow continuously without overfeeding or starvation, respectively, based on a balance of glucose release and consumption. The changes of cell densities were reflected very well by the oxygen profile (Figure 3c). For example, with more biocatalyst cell densities were always higher and oxygen contents were always lower in the first 12 h of cultivation. Finally, all cell densities reached high OD_600_ values of ~20, although without addition of complex nutrient additives which were used in our previous studies [18]. With a biocatalyst concentration of 3 U L^−1^, valinomycin reached the highest titer of nearly 800 μg L^−1^ and the corresponding yield was the second highest of 63 μg g^−1^ biomass (Figure 3d). In addition, the pH values remained stable at ~7.1 under this condition, which is more close to the optimal pH of 7.0 for *E. coli* growth. Therefore, 3 U L^−1^ of biocatalyst was applied to our following scale-up bioprocess development.

### Shake flask cultivation

**Figure 4:**
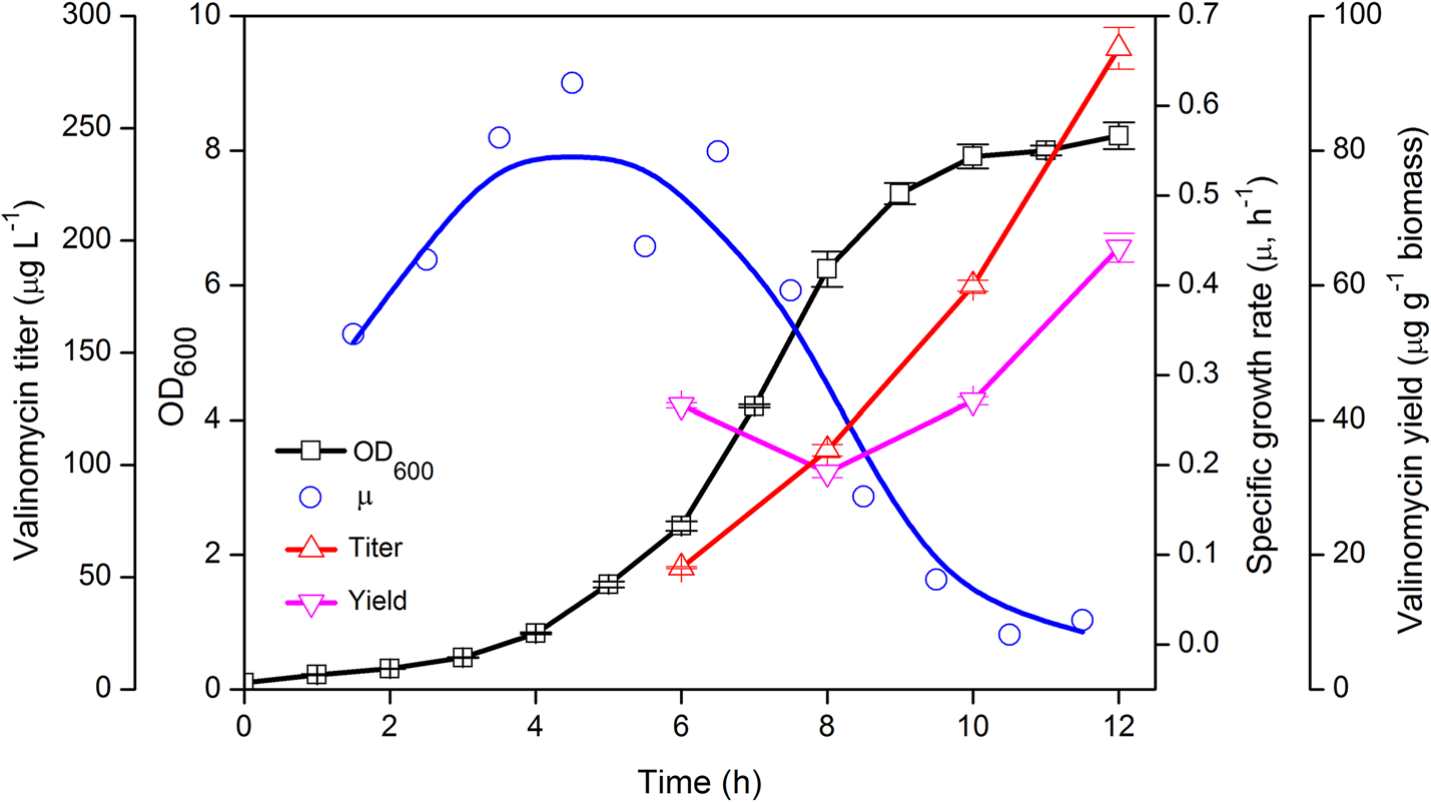
Valinomycin production in shake flask scale cultivations of *E. coli* BJJ01 pCTUT7-Vlm1 pKS01-Vlm2. Cultivations were performed in 500 mL flasks containing 100 mL EnPresso B defined medium at 200 rpm and 30°C for 12 h. The glucose releasing biocatalyst concentration was 3 U L^−1^.

Before moving forward to bioreactor cultivations, we aimed to figure out the correlations between the specific growth rate of the culture and the rate of valinomycin formation, which could likely be referred for the bioreactor feeding strategy. To this end, we conducted cultivations in shake flasks using EnPresso B defined medium supplemented with 3 U L^−1^ of biocatalyst. As shown in Figure 4, after an initial lag phase cells experienced an exponential growth with a maximum specific growth rate (*μ*) of appr. 0.55 h^−1^, which declined after 5–6 h slowly to the end of the cultivation. Since leaky expression plasmids were used in our system, no IPTG was added to induce enzyme (Vlm1 and Vlm2) expression. Therefore, Vlm1 and Vlm2 can be gradually expressed from the beginning of the cultivation to synthesize valinomycin, and after 6 h cultivation the valinomycin titer was determined to be around 50 μg L^−1^. Although the *μ* dropped below 0.2 h^−1^ between 8 and 12 h, valinomycin accumulated up to approximately 300 μg L^−1^ with the highest specific product formation rate (*q*_p_) of 6.87 μg L^−1^ OD^−1^ h^−1^ during this period. This could be probably explained that cells grow slower, but have enough time to express the two large enzymes for the biosynthesis of valinomycin. On the other hand, increasingly expressed enzymes and valinomycin can improve cell metabolic burden and thus inhibit cell growth. The results indicate that the accumulation of valinomycin could be maintained even at a specific growth rate below 0.2 h^−1^. Moreover, shake flask cultivations also generated similar valinomycin yields (65 μg g^−1^ biomass) compared to the 24-well plate cultivations (63 μg g^−1^ biomass). From these experiments we concluded that the formation of valinomycin is relatively robust in relation to the specific growth rate and may even be higher at low growth rates.

### Bioreactor high cell density fed-batch fermentation

In order to scale up the cultures to higher cell densities, a cultivation was performed in a 3.7 L bench-top bioreactor with 2 L culture volume. Instead of the commonly used initial batch mode cultivation in the bioreactor, the fed-batch-like EnPresso B was used as a starter medium for the high cell density cultivation, which would confer cells with consistent intracellular physiological conditions, avoiding overflow metabolism in the initial cultivation phase [24]. Since modest yields of valinomycin could be reached without nutrient boosting (Figure 3d), the bioreactor fed-batch production was performed only based on pure glucose feeding without addition of complex nutrient additives. The cultivation in the bioreactor was started with an initial OD_600_ of 0.1 and 3 U L^−1^ of biocatalyst was added for glucose release in the initial phase. During the whole fermentation, *E. coli* BJJ01 pCTUT7-Vlm1 pKS01-Vlm2, the same strain as in the previous small scale cultivations, was grown at 30°C with a constant pH of 7.0 and the DO was maintained above 20%.

**Figure 5:**
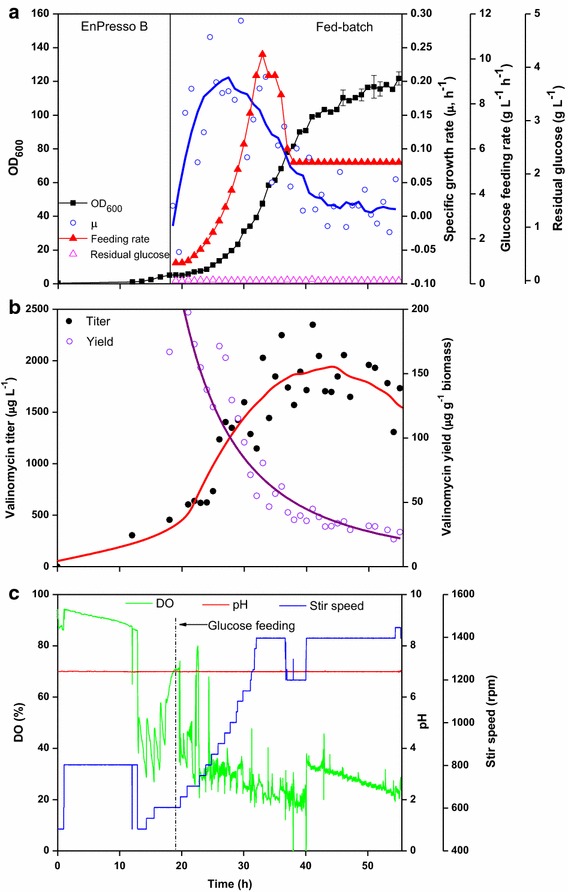
Fed-batch bioreactor cultivation for valinomycin production. **a** Cell growth curve, specific growth rate, glucose feeding rate and residual glucose concentration; **b** valinomycin production; **c** profiles of online dissolved oxygen (DO), pH and stir speed.

After 16–18 h of cultivation, the glucose polymer in the EnPresso B medium was depleted, as indicated by the continuous increase of DO (Figure 5c). Also the levels of glucose were below the detection limit in this period (Figure 5a). Therefore, at 19 h the external glucose feeding was initiated with an exponential feed profile for a targeted specific growth rate (*μ*) of 0.2 h^−1^. The feeding was continued for 12 h followed by a gradual decrease to a constant feeding to the end of the fermentation (Figure 5a) to avoid the culture to run into oxygen limitation.

Initially, cells grew exponentially along with the exponential feeding protocol and reached the specific growth rate of 0.2 h^−1^. When the feeding was adjusted to a constant rate, the specific growth rate dropped to below 0.05 h^−1^ until the end of the cultivation with a final OD_600_ of approximately 120 (Figure 5a).

**Figure 6:**
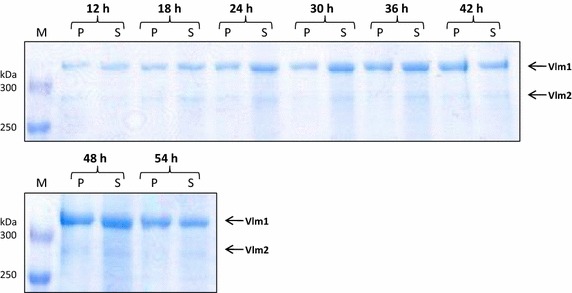
SDS-PAGE analysis of Vlm1 and Vlm2 expression during the bioreactor fermentation. Cells were collected at different time intervals from the bioreactor as shown on the *top* of the gels. Protein samples were separated on a 5% polyacrylamide gel. Vlm1 (370 kDa) and Vlm2 (284 kDa) are indicated by *arrows*. *P* insoluble protein, *S* soluble protein, *M* protein molecular weight marker.

Valinomycin titers were measured hourly after starting of the glucose feeding (see Figure 5b). The volumetric valinomycin concentration increased during the exponential feed phase to a titer of about 2 mg L^−1^ followed by a gradual slight decrease, which is due to the continuous dilution of the bioreactor by the feed solution. The valinomycin yield based on biomass was the highest of appr. 150 μg g^−1^ biomass until the mid of the exponential feed phase but declined in the second half of the cultivation. Unlike our previous results that high cell densities generated high valinomycin concentration in the shake flask scale [18], a high cell density cultivation in the bioreactor did not further improve the valinomycin titers. SDS-PAGE analysis of Vlm1 and Vlm2 bands showed a distinct difference to all our previous cultivations, where both enzymes were expressed approximately with the same strength. While Vlm1 was expressed to a high level throughout the whole fermentation, Vlm2 was not well expressed (Figure 6). The much lower expression of Vlm2 is probably due to the plasmid instability in the cells because pCTUT7-Vlm1 and pKS01-Vlm2 have the same origin of replication. Interestingly enough, the expression of Vlm2 had not been a problem in our earlier studies at the small scale (see also [17, 18]). Therefore, we decided in the following scale up study to use the strain with plasmids which contain different origins of replication as described in our recent paper [19].

### Valinomycin production in a two-compartment reactor

While the previous fed-batch bioreactor process indicated that the scale up of the valinomycin process from shaken cultures to bioreactor scale is not easily predictable, we aimed to test the robustness of valinomycin production under conditions which represent a larger industrial scale.

Therefore, parallel experiments were performed either in a stirred tank reactor (STR) or in a scale-down TCR (see Figure 2d). According to the results we had two hypotheses: (1) the high glucose levels and oxygen limitation in the plug flow reactor (PFR) part of the reactor together with the higher starvation effect in the STR compartment might decrease the valinomycin production; (2) while previous investigations indicated that pyruvate and l-valine can be significantly accumulated in *E. coli* after a repeated short-time downshift of oxygen at a high glucose concentration in a bioreactor fed-batch fermentation [30] and also under repeated oscillations of these conditions in a scale down bioreactor [31], one also might hypothesize that such a two-compartment bioreactor cultivation could even increase valinomycin biosynthesis through increasing the flux of glucose to the synthesis of its precursors pyruvate, α-ketoisovalerate and l-valine. To simulate these conditions, we used a 15 L scale-down TCR (see Figure 2d) for valinomycin production. This TCR system allows continuous circulation of the culture between a STR and a PFR, generating the oscillating fermentation conditions with high glucose and oxygen limitation in the PFR loop with a residence time of approximately 1 min [26, 27].

**Figure 7:**
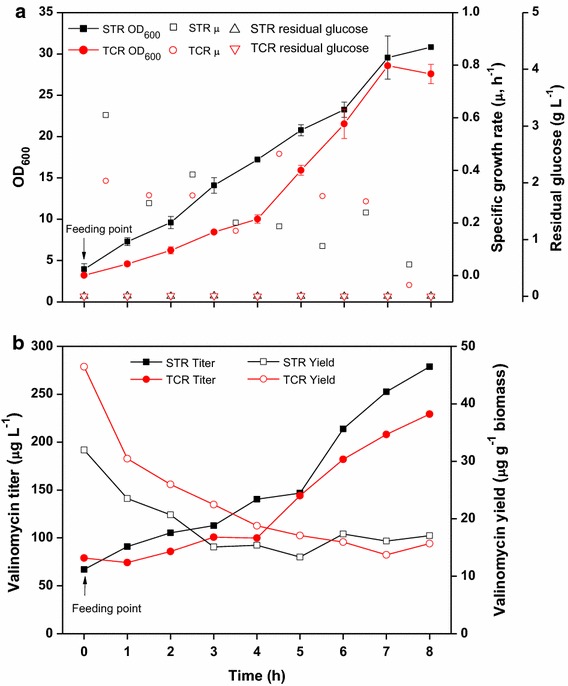
Valinomycin production under oscillating conditions in a scale-down two-compartment reactor. **a** Cell growth curves, specific growth rate and residual glucose concentration; **b** valinomycin production. *STR* stirred tank reactor, *TCR* two-compartment reactor.

**Figure 8:**
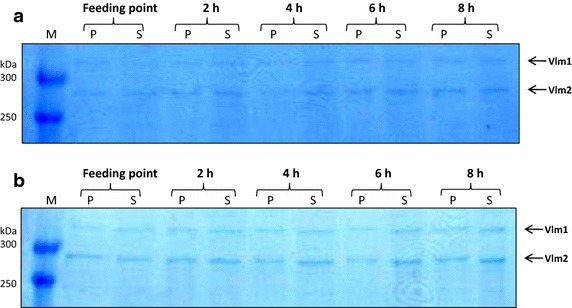
SDS-PAGE analysis of Vlm1 and Vlm2 expression during the two-compartment bioreactor fermentation. **a** Vlm1 and Vlm2 expression in the STR; **b** Vlm1 and Vlm2 expression in the TCR. Cells were collected at different time intervals as shown on the *top* of the gels. Protein samples were separated on a 5% polyacrylamide gel. Vlm1 (370 kDa) and Vlm2 (284 kDa) are indicated by *arrows*. *P* insoluble protein, *S* soluble protein, *M* protein molecular weight marker.

To overcome the instability of multiple plasmids in one cell, we have constructed new plasmids with compatible origins of replication for enzyme expression, and we further improved the system by coexpression of the TEII enzyme [19]. Figure 7 shows valinomycin production with the strain *E. coli* BJJ01 pJL03-Vlm1 pJL07-Vlm2 pJL10-TEII under oscillating conditions in the scale-down TCR (STR + PFR) system. The fermentation in the STR without PFR was performed as a reference. In both the STR and the TCR fermentations, glucose feeding was initiated at an OD_600_ of ~4 (Figure 7a). In the STR, glucose was directly fed into the bioreactor, while in the TCR glucose was fed at the inlet of the PFR (see Figure 2d), forming a high glucose and anaerobic growth environment. The feeding process was continued for 8 h and samples were drawn from the STR every hour for analysis. In agreement with published data, OD_600_ values in the control STR fermentation were always higher than those in the TCR fermentation even though the OD_600_ values were similar at the starting point of glucose feeding (Figure 7a). This is typical for all large scale processes with imperfect mixing [27]. Likewise, the volumetric valinomycin titers in the TCR fed-batch fermentation were also lower than the reference STR fermentation (Figure 7b). However, the specific valinomycin yields (i.e. g per g cell biomass) were higher in TCR than in the STR fermentation during the initial 6 h fed-batch cultivation (Figure 7b). The expression levels of Vlm1 and Vlm2 were analyzed by SDS-PAGE and as expected both enzymes were expressed equally without a significant change over the cultivation (Figure 8).

This first result is promising, as it indicates that valinomycin production is not disturbed by effects which are caused by non-perfect mixing and therefore the process should be robust for scale up. Furthermore, the higher yield of valinomycin on a cell basis (Figure 7b) suggests that there is space for further optimization by variation of the perturbances. Possibilities are variations in the strength and the intervals of the pulses. Such engineering approaches should go hand in hand with a further optimization of the genetic system. The biomass specific production yields for the strain *E. coli* BJJ01 pJL03-Vlm1 pJL07-Vlm2 pJL10-TEII are only 30–50% of the production yields in *E. coli* BJJ01 pCTUT7-Vlm1 pKS01-Vlm2, which well agrees with the higher amounts of soluble Vlm1 and Vlm2, respectively, in the latter strain (data not shown). Thus, we conclude that also the amount of Vlm1 and Vlm2 currently limits the accumulation rate of valinomycin.

## Conclusions

In this work, valinomycin production was scaled up from mL volumes to 10 L scale with consistent fed-batch cultivations, which illustrates a blueprint of rational bioprocess development for other complex natural product production. Based on the fed-batch-like EnBase cultivation system, the process development procedure for valinomycin production from small to larger scale was efficient by the ability to perform simple-to-perform parallel cultivations. The bioreactor high cell density fed-batch fermentation produced valinomycin with titers of more than 2 mg L^−1^ based on a pure glucose feeding strategy. Valinomycin production was not negatively affected by the oscillating conditions (i.e. glucose supply and DO) in a scale-down TCR that mimics similar situations in industrial bioreactors, indicating that a scaling of the bioprocess to large industrial scale should be achievable.
